# Cannibalism Affects Core Metabolic Processes in *Helicoverpa armigera* Larvae—A 2D NMR Metabolomics Study

**DOI:** 10.3390/ijms17091470

**Published:** 2016-09-02

**Authors:** Fredd Vergara, Amiu Shino, Jun Kikuchi

**Affiliations:** 1 RIKEN Center for Sustainable Resource Science, 1-7-22 Suehiro-cho, Tsurumi-ku, Yokohama 230-0045, Japan; amiu.shino@riken.jp; 2 Graduate School of Medical Life Science, Yokohama City University, Yokohama 230-0045, Japan; 3 Graduate School of Bioagricultural Sciences, Nagoya University, Nagoya 464-8601, Japan

**Keywords:** cannibalism, *Helicoverpa armigera*, HSQC, NMR, metabolomics

## Abstract

Cannibalism is known in many insect species, yet its impact on insect metabolism has not been investigated in detail. This study assessed the effects of cannibalism on the metabolism of fourth-instar larvae of the non-predatory insect *Helicoverpa armigera* (Lepidotera: Noctuidea). Two groups of larvae were analyzed: one group fed with fourth-instar larvae of *H. armigera* (cannibal), the other group fed with an artificial plant diet. Water-soluble small organic compounds present in the larvae were analyzed using two-dimensional nuclear magnetic resonance (NMR) and principal component analysis (PCA). Cannibalism negatively affected larval growth. PCA of NMR spectra showed that the metabolic profiles of cannibal and herbivore larvae were statistically different with monomeric sugars, fatty acid- and amino acid-related metabolites as the most variable compounds. Quantitation of ^1^H-^13^C HSQC (Heteronuclear Single Quantum Coherence) signals revealed that the concentrations of glucose, glucono-1,5-lactone, glycerol phosphate, glutamine, glycine, leucine, isoleucine, lysine, ornithine, proline, threonine and valine were higher in the herbivore larvae.

## 1. Introduction

Cannibalism is the process of feeding on parts of or whole individuals of the same species [1]. This phenomenon is taxonomically widespread but uncommon in most animal taxa [2]. Cannibalism may be advantageous since feeding on individuals of the same species, i.e., conspecifics, may represent a source of high quality nutrients. It could also eliminate competitors and predators, thereby making available valuable ecological resources [3,4]. If cannibalism produces such positive effects it could be expected to occur more commonly than it actually does. Perhaps negative effects associated with cannibalism outweigh the benefits under some circumstances, or for some species cannibalism is intrinsically not as beneficial as it is assumed. When ingesting conspecifics, cannibals expose themselves to injuries from prey that fight back and to the pathogens and parasites present in the victim [2,5]. Cannibalism may also reduce the chances of finding a mate. However, it could also be possible that conspecifics simply do not represent the best quality diet for some animal species.

Cannibalism has been reported in many insect species, both carnivores and herbivores. One example is the larval stages of *Helicoverpa armigera* (*H. armigera*, Hübner, Lepidotera: Noctuidea), the cotton bollworm, when kept under laboratory conditions [6]. *H. armigera* is one of the worst insect pests of agriculture worldwide, attacking a wide range of food, fiber, oil, and fodder crops as well as many horticultural and ornamental crops. It thereby causes economic damage amounting annually to millions of dollars in crop losses and environmental damage due to the extensive use of insecticides required to control its populations [7,8]. Cannibalism in Lepidoptera is commonly associated with environmental factors such as high population densities but it may also have a genetic component. In *Helicoverpa virescens*, a close relative of *H. armigera*, inheritance has been shown to determine different levels of cannibalism over a geographic range [3]. Due to its economic and ecologic relevance, *H. armigera* has become a model system for entomology, yet the impact of cannibalism on its metabolism has not been evaluated. Therefore, in the present study it was investigated how cannibalism shapes the *H. armigera* metabolism and influences its development. *H. armigera* larvae were fed with conspecifics of the same size (henceforth “cannibals”) or with an artificial plant diet (“herbivores”). It was expected that if conspecifics represent a good quality diet, the growth rate and survival of cannibals and herbivores should be at least similar. It was also examined whether cannibals and herbivores had similar metabolic fingerprints, which were determined by two-dimensional nuclear magnetic resonance.

## 2. Results and Discussion

**Figure 1:**
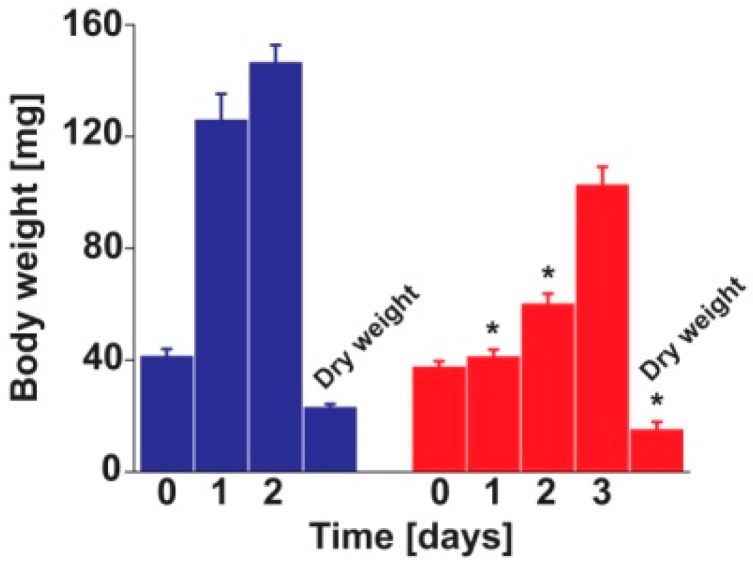
Growth rate of herbivore (blue) and cannibal (red) *Helicoverpa armigera* larvae. Weight of individual larvae was recorded from the moment a group of larvae was switched from herbivore diet to cannibalism. Larvae were in third instar at the beginning of the experiment. Bars represent mean and standard error of the mean; *t*-tests were computed to identify differences between diets at specific days (* *p* > 0.05).

*Helicoverpa armigera* cannibals were smaller than the herbivores (Figure 1), a result in agreement with previous reports [6]. The present study, however, pioneers in finely dissecting the consequences of cannibalism on the physiology of this insect. It is not known how often *H. armigera* larvae cannibalize each other in nature nor if there are larvae that are entirely cannibalistic or if larvae actually switch back and forth between herbivory and cannibalism. However, this study indicates that pure cannibalism has a negative impact on the development of *H. armigera*. Previous reports also showed that, under laboratory conditions, cannibalism negatively affected pupation with cannibals showing a six-fold decrease in successful pupation [6]. If this negative effect on pupation also exists in nature, it is not known. In this study no anatomical differences associated with size differences were observed between cannibals and herbivores. Strong differences were detected, however, in the ^1^H-^13^C HSQC (Heteronuclear Single Quantum Coherence) spectra of larval D_2_O extracts. NMR metabolomics studies are vastly dominated by ^1^H spectroscopy for the profiling of samples (Figure S1), with ^1^H-^13^C HSQC being the preferred technique for compound identification. This is mainly due to the higher sensitivity of ^1^H spectroscopy; however, ^1^H-^13^C HSQC can also be used for the profiling itself. The inherent disadvantage in low sensitivity is compensated by the increment in the number of signals detected per sample as a result of the spreading of the ^1^H signals on the ^13^C dimension [9,10]. Signals in the ^1^H-^13^C HSQC spectra were manually defined as 135 regions of interest (ROIs) using rNMR (Figure S2). The chemical shifts of the 135 ROIs (the centroid of the ^1^H-^13^C cross-peaks) were analyzed with SpinAssign to generate a list of candidate metabolites present in the extracts (Table S1). The chemical shifts of the candidate metabolites were confirmed using two additional public databases (HMBD and BMRB).

**Figure 2:**
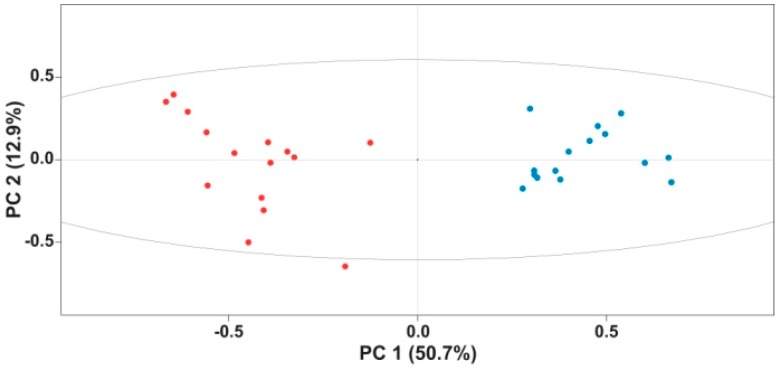
Principal component analysis (PCA) score plot of ^1^H-^13^C HSQC regions of interest (ROIs). D_2_O extracts of herbivore (blue dots) and cannibal (red dots) *Helicoverpa armigera* larvae formed two distinct groups.

**Figure 3:**
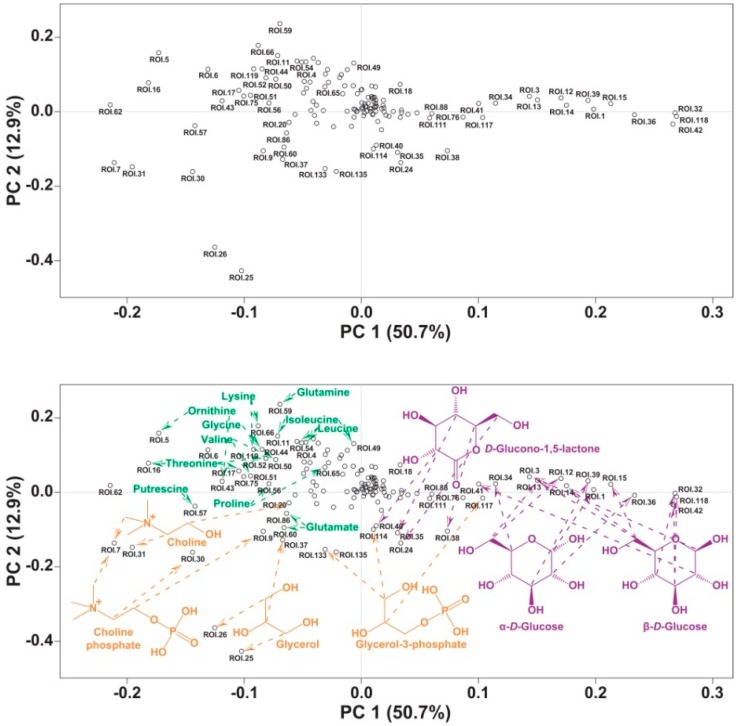
PCA-loading plot of ^1^H-^13^C HSQC ROIs. **Upper** panel: Raw loadings chart; **Lower** panel: structures of identified metabolites were added. Notice that in several cases a single metabolite produced multiple ROIs.

In parallel, the 135 ROIs were batch integrated (all the ROIs for all the spectra at once) in rNMR to generate a matrix of signal integrals (Table S2). This ROI data matrix was subjected to principal component analysis (PCA). PCA scores showed a distinct signature for the chemical profiles of cannibal larvae, with the formation of two separated clusters along PC1 (Figure 2), a pattern also displayed when ^1^H NMR data obtained from the same samples were used for PCA (Figure S3, Table S3). Interestingly, within-group variation along PC2 was larger in the cannibals. Since all larvae used for the assay were randomly chosen from the main population, it is unexpected that this phenotypic difference has a genetic component; rather, it must be caused by an environmental factor. The difference in variation along PC2 might be the result of the higher homogeneity of the plant diet ingested by the herbivores. All larvae, cannibals and herbivores, were provided with an amount of food larger than they could eat to prevent metabolic differences caused by starvation. It is possible that some cannibals ingested more heads while other cannibals ingested proportionally more thoraxes or abdomens, generating more variation in the metabolic profiles of cannibals. The combination of the PCA loadings with the identification of the metabolites producing the different ROIs showed that several of the metabolites with the largest variability were grouped into three classes: monomeric sugars, fatty acid-related compounds and amino acids and amino acid-related compounds (Figure 3).

**Figure 4:**
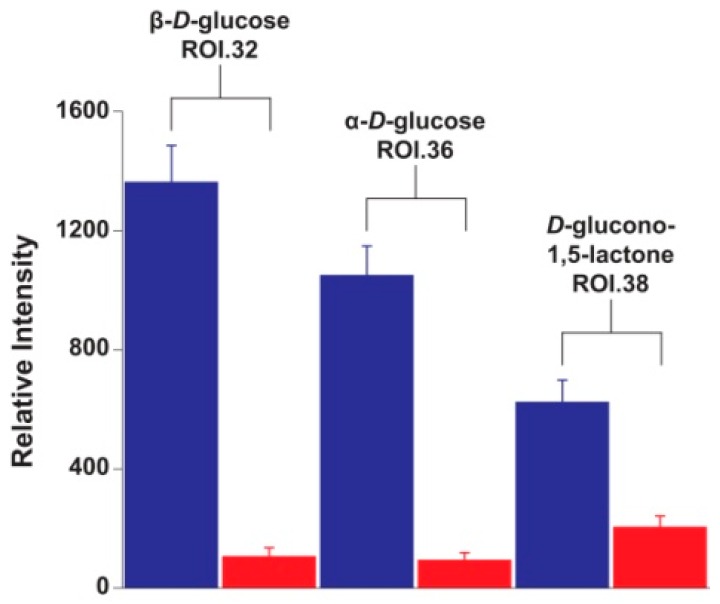
Quantitation of monomeric sugars in D_2_O extracts of *Helicoverpa armigera* larvae. Quantitation was performed on a single ^1^H-^13^C HSQC ROI per metabolite. ROIs with the highest variability according to PCA loadings were selected. Herbivores = blue; cannibal = red.

HSQC data are primarily used for qualitative purposes, yet direct relative quantitation of HSQC signals is possible due to the intrinsic highly linear response of this NMR experiment [11]. Thus, in order to have a clearer view on the variation of individual metabolites among diets, the integrals of one ROI per metabolite were compared in cannibal and herbivore larvae. If a given metabolite produced more than one ROI, then ROIs that did not overlap with other metabolites were used for intensity comparison. Using this approach it was observed that the largest difference in concentration for all metabolites was detected for d-glucose which was much more abundant in the herbivore larvae (Figure 4). The other identified monomeric sugar, d-glucono-1,5-lactone, was also more abundant in herbivores (Figure 4). d-glucono-1,5-lactone is an oxidation product of d-glucose. The reaction is carried out by glucose oxidase, an enzyme secreted in the labial salivary glands of *H. armigera* [12].

**Figure 5:**
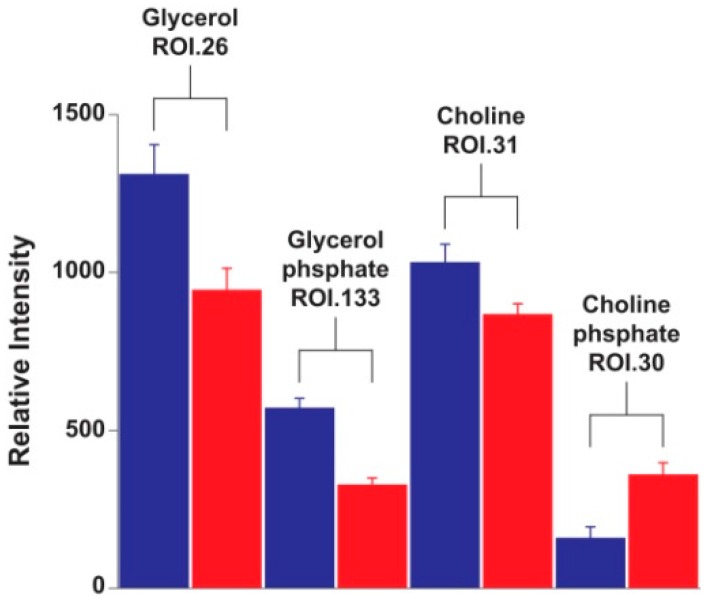
Quantitation of fatty acid-related metabolites in D_2_O extracts of *Helicoverpa armigera* larvae. Quantitation was performed on a single ^1^H-^13^C HSQC ROI per metabolite. ROIs with the highest variability according to PCA loadings were selected. Herbivores = blue; cannibals = red.

In the case of the fatty acid-related metabolites, glycerol phosphate was much more abundant in the herbivores (Figure 5). Also more abundant in the herbivores were glycerol and choline; however, choline phosphate was more abundant in cannibals (Figure 5). Choline belongs to a special class of nutrients termed “quasi-vitamins” or “conditionally essential” nutrients and its absence from artificial diets results in a somewhat reduced insect growth rate [13]. Choline is absorbed by the midgut cells of *Pieris brassicae*, a lepidopteran like *H. armigera*. It is then transported into the hemolymph and later converted, probably in the fat body, into phospholipids [14]. The phosphate ester of choline, choline phosphate, is the most abundant choline metabolite in the hemolymph of the silkworm (*Bombyx mori* L., also a lepidopter) and its concentration increases as the larvae approaches pupation [15]. Choline phosphate is involved in the biosynthesis of phospholypids for the formation of cell membranes.

**Figure 6:**
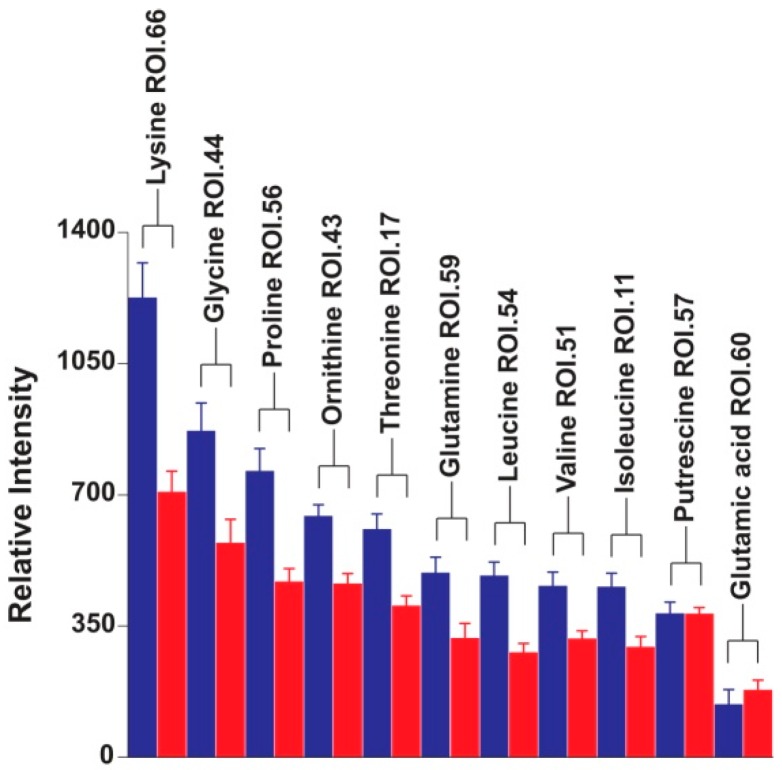
Quantitation of amino acids and amino acid-related metabolites in D_2_O extracts of *Helicoverpa armigera* larvae. Quantitation was performed on a single ^1^H-^13^C HSQC ROI per metabolite. ROIs with the highest variability according to PCA loadings were selected. Herbivores = blue; cannibal = red.

As for the amino acids and amino acid-related compounds, they were more abundant in herbivores, with the exception of glutamate, which was slightly more abundant in cannibals, and putrescine, which showed comparable concentrations in cannibals and herbivores (Figure 6). Putrescine has been shown to have a high incidence during diapause in *H. armigera* [16]. Diapause is a programmed developmental arrest coupled with the depression of metabolic activity, and in *H. armigera* diapause induction and preparation occur in the larval stage [17]. Thus, the fact that cannibals and herbivores contained the same amount of putrescine implies that both types of caterpillars in this study were found in a comparable developmental stage. This reinforces the fact that the observed differences in the metabolic profiles of cannibals and herbivores are the result of the diet and not the larval ontology.

Some evidence considers cannibalism as adaptive [1,18,19,20], and in the case of herbivore insects feeding on conspecifics, it may provide nutrient compositions that are more optimal than plant diets. For herbivores, conspecific predation may provide a meal with a higher carbon-to-nitrogen ratio (C:N ratio) than the normal plant diet and it has been suggested that cannibalism in such animals may result from a specific need for protein [21]. On the other hand, there is evidence that cannibalism has a negative impact on insect fitness. For instance, in larvae of the lepidopteran *Spodoptera frugiperda*, cannibalism reduces survival and pupal weight [22], and larval cannibalism in the lepidopteran *Ascia monuste orseis* produces smaller adults [23]. Considering the contrasting evidence, it is possible that cannibalism may have positive or negative effects, depending on the species involved and the environmental conditions. The present study with its pioneering nature provides a strong case for depletion in nutrient availability as a possible cause for the negative consequences of cannibalism observed in some insect species. Additionally, not only the total amount of but the ratios between different classes of nutrients can be critical in explaining the performance of cannibals. Particularly, the carbohydrate-to-protein ratio has been shown to be critical for the performance (growth rate × survival) of the orthopteran *Chortoicetes terminifera* [24]. In this species a 2:1 carbohydrate-to-protein ratio has been found as an optimal diet. If the amount of free amino acids that we detect in the bodies of *H. armigera* larvae at least partially reflects the amount of protein ingested, then the carbohydrate-to-protein ratio would be larger in the herbivores and this might be connected to their larger size. Furthermore, the carbohydrate-to-protein ratio in the insect diet influences the absorption efficiency of carbohydrates and proteins in the insect gut and this has been correlated with specific gut microbiotas and the functionality of the insect immune system [24] and this, in turn, could also explain the differences in the performance between herbivore and cannibal *H. armigera* larvae.

## 3. Materials and Methods

### 3.1. Insect Bioassay

*Helicoverpa armigera* eggs were purchased from Sumika Technoservice Corporation (Terazuka-shi, Hyogo, Japan). Larvae were fed from hatching with the artificial diet Silk Mate 2S based on mulberry leaves and manufactured by Nosan Corporation (Yokohama-shi, Kanagawa, Japan). Upon arrival to the laboratory larvae were kept in 2 mL Eppendorf tubes, one larva per tube with plenty of artificial diet. Once the larvae reached an average weight of 40 mg thirty larvae were transferred to Petri dishes, one larva per dish. Third- to fourth-instar larvae (aprox. 40 mg) were used because cannibalism is less common in earlier [6] and latter [25,26] larval stages of *H. armigera* in captivity and also to maximize the amount of sample available per larva for NMR analyses. Larvae used for the bioassay were randomly chosen from the main stock. Fifteen larvae were fed with the Silk Mate 2S diet (herbivore group) and the other 15 larvae (cannibal group) were fed with prey larvae from the main stock (larvae always fed with artificial diet). Larvae used as prey were selected to have a similar size as the cannibals they were going to feed. The jaws of the prey larvae were disabled by longitudinally splitting the larva’s head using a scalpel, thereby preventing wounds caused by prey larvae fighting back. Every cannibal larva was offered one live, disabled prey larva in the morning and one more in the afternoon to ensure that cannibals were never devoid of food. The remains of the previous prey larva were always removed when placing the new one in the Petri dish to minimize the risk of infection for the cannibals. Insects were kept at 28 °C under natural light. After two days in the case of the herbivores and three days for the cannibals, larvae were killed by placing them at −20 °C. Larvae were sacrificed when they started showing signs of preparation for pupation, which happened at different times between diets. Whole larvae were freeze-dried and the water-free material was weighed and used for NMR analyses. The Ministry of Education, Culture, Sports, Science and Technology of Japan does not require ethical approval for working with insects. All efforts were made to minimize suffering of the larvae.

### 3.2. Sample Preparation for NMR

To minimize chemical shift variations due to pH differences across samples a 1 mM phosphate buffer (KPi) was used as solvent. It consisted of 10.7 g K_2_HPO_4_ + 5.24 g KH_2_PO_4_ in 1 L D_2_O (D 99.9% Cambridge Isotope) containing 1 mM DSS-*d*_6_ (D 98% Cambridge Isotope) as internal standard. The buffer was directly measured with a pH-meter, which showed a value (pD) of 7.0. Experimental and theoretical approaches showed that pH = pD + 0.41 [27], thus the pH of the buffer was 7.41. Freeze-dried larvae were individually placed in 1.5 mL Eppendorf tubes and covered with 700 µL buffer. Larvae were ground-up with a plastic pestle. Samples were centrifuged at 17,500× *g* at 20 °C for 10 min. After recovering the supernatant dilutions were made against the larva with the smallest dry weight in the whole experiment in order to normalize the amount of insect material per sample. The final volume for all samples was 600 µL of deuterated solvent.

### 3.3. NMR Spectra Acquisition and Processing Parameters

Spectra were recorded using an Avance II 700 Bruker spectrometer equipped with a 5 mm inverse CryoProbe operating at 700.153 MHz for ^1^H and 176.061 MHz for ^13^C. Acquisition temperature was 298 K. The ^1^H spectra were acquired using a water suppression pulse program with the following conditions: 0.38 Hz × point^−1^, acquisition time = 1.31 s, relaxation delay = 2.0 s and 90° pulse width = 8 µs. Sixty-four transients with 0 dummies were recorded per spectrum. FIDs (free induction decays) were Fourier-transformed using line broadening (LB = 1.0 Hz). The resulting spectra were manually phased and baseline-corrected and calibrated to the internal standard (DSS-*d*_6_). ^1^H-^13^C HSQC spectra were acquired using the Bruker pulse program hsqcetgp for echo/antiecho gradient with the following conditions: relaxation delay = 2.0 s; 7043 Hz spectral width in f1 and 9804 Hz in f2; 90° pulse widths = 10 µs for hydrogen, 15 µs for carbon. The partial spectral width (aliasing) in the indirect dimension (40 ppm in ^13^C) allowed to reduce the acquisition time per spectrum without losing spectral resolution [28,29]. Qsine (SSB = 2.0) was used for the window function. Sixteen dummies and 64 transients were collected. FIDs were Fourier-transformed and the resulting spectra were manually phased, baseline corrected and calibrated using glucose (C1: δ^1^H = 5.22376, δ^13^C = 54.764) and allantoate (δ^1^H = 5.23749, δ^13^C = 62.8906) signals.

### 3.4. Multivariate Statistical Analyses of ^1^H and ^1^H-^13^C HSQC NMR Spectra

^1^H NMR spectra. Phased and baseline-corrected ^1^H NMR spectra were binned from 10.0 to 0.5 ppm with a bin width of 0.02 ppm using the software automics [30]. Binning was performed applying the total scaling function of automics and the region from 5.0 to 4.6 ppm (HDO signal) was not binned. The matrix with the data of all the binned spectra (455 bins per spectrum) was exported to the software R (r-project.org) and processed with the package muma for NMR metabolomics [31]. Principal component analysis (PCA) was performed according to muma’s manual and applying the following parameters: scaling = “pareto”, imput = “mean”, normalize = TRUE. Pareto scaling was chosen as the data stays closer to the original measurement.

^1^H-^13^C HSQC NMR spectra. Phased, baseline-corrected and calibrated ^1^H-^13^C HSQC NMR spectra were processed with rNMR (http://rnmr.nmrfam.wisc.edu/) to manually generate a list of regions of interest (ROI) with signals present across all samples. One hundred thirty-five ROIs were identified and the signals intensities in every ROI were obtained in batch mode with the following parameters: type = area, normalization = signal to noise. The matrix of ROIs intensities was exported to the software R and processed with the package muma for NMR metabolomics [30]. Principal component analysis (PCA) was performed according to muma’s manual and applying the following parameters: scaling = “pareto”, imput = “mean”, normalize = TRUE.

### 3.5. Candidate Compounds

The ^1^H-^13^C cross-peak chemical shifts were recorded at the center of each ROI to generate a list of chemical shifts that was exported to the databases SpinAssign (http://prime.psc.riken.jp), HMBD (http://www.hmdb.ca/) and BMRB (http://www.bmrb.wisc.edu/metabolomics/) for compound identification by signal matching.

## 4. Conclusions

We present the first evidence of the dramatic changes in body chemical composition experienced by *Helicoverpa armigera* larvae when they become completely cannibalistic and the negative effect that this has on the growth of cannibals, and we elaborate on how these changes might shape the fitness of this insect.

## Supplementary Materials

Supplementary materials can be found at www.mdpi.com/1422-0067/17/9/1470/s1.
