# Supplementary material for: Cannibalism Affects Core Metabolic Processes in Helicoverpa armigera Larvae—A 2D NMR Metabolomics Study
Source: Int J Mol Sci. 2016 Sep 2;17(9):1470. doi: 10.3390/ijms17091470 (PMC5037748; doi:10.3390/ijms17091470)
Supplement: Supplementary file 1 [file ijms-17-01470-s001.zip › ijms-131501-Supplementary Materials/ijms-131501-supplementary.pdf]

Supplementary Materials: Cannibalism Affects Core Metabolic Processes in *Helicoverpa armigera* Larvae—A 2D NMR Metabolomics Study

Fredd Vergara, Amiu Shino and Jun Kikuchi

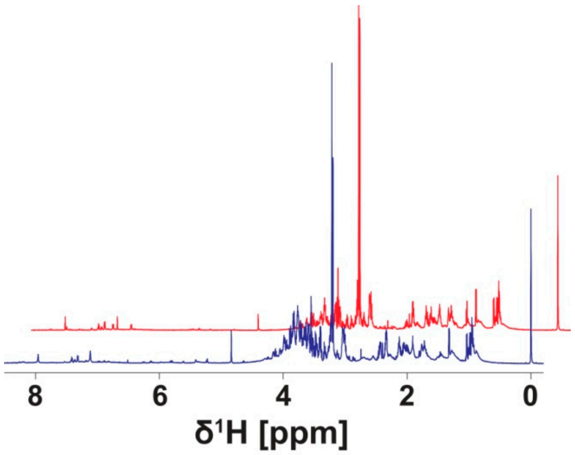

Figure S1. Representative ^{1}H spectra. Cannibals (red) and herbivores (blue) are offset to facilitate visualization.

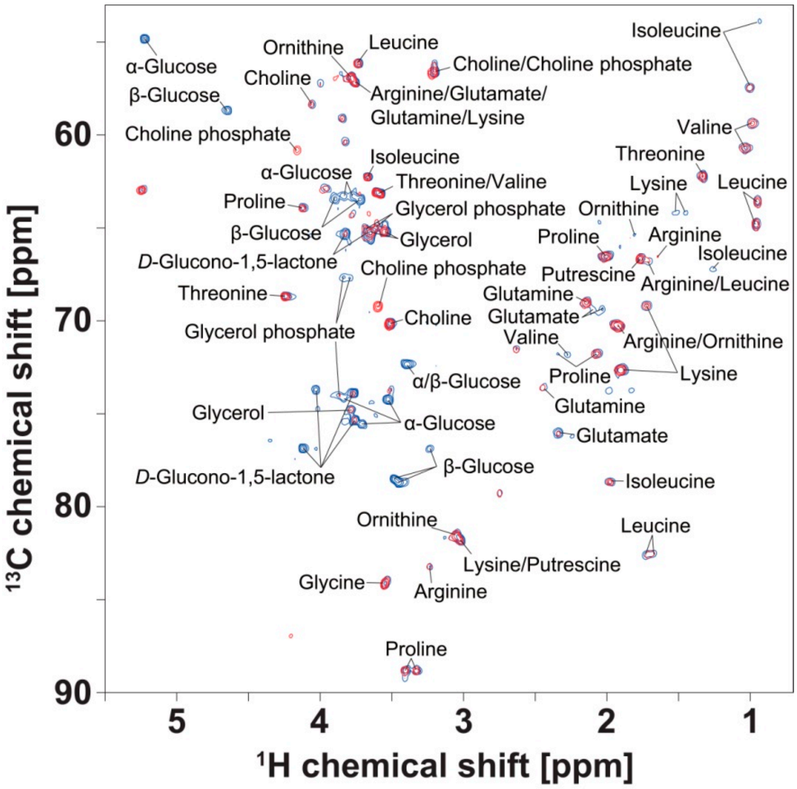

Figure S2. Representative ^{1}H-^{13}C HSQC spectra. Cannibals (red) and herbivores (blue) signals are overlaid for direct comparison. Notice that as a consequence of the narrow spectral width in the indirect dimension (carbon), signals of some metabolites are aliased (offset) 40 ppm (see Materials and Methods section).

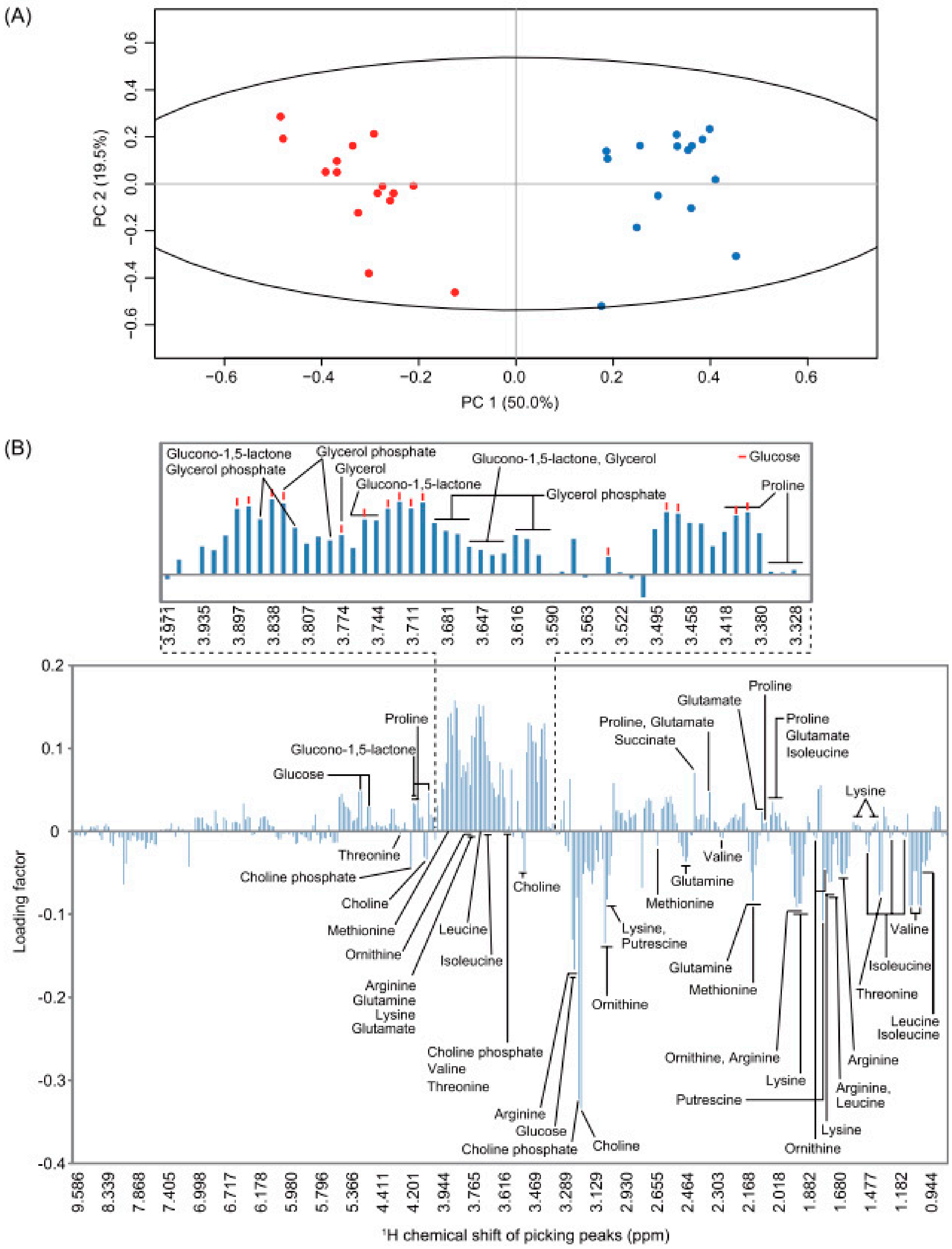

Figure S3. PCA score plot of peak-aligned ^{1}H spectra (**A**). D_2O extracts of herbivore (blue dots) and cannibal (red dots) *Helicoverpa armigera* larvae formed two distinct groups as in ^{1}H-^{13}C HSQC ROIs (Figure 3). Notice that ^{1}H and ^{1}H-^{13}C HSQC spectra were obtained from the same larval extracts. PCA-loading plot of peak-aligned ^{1}H spectra (**B**).

Table S1. List of candidate metabolites by matching of the ^{1}H and ^{13}C chemical shifts (obtained from HSQC spectra) using SpinAssign and confirmed by comparison with the databases HMDB and BRMB. Notice that as a consequence of spectral aliasing, some signals appear offset by 40 ppm. ROIs used to produce Figures 2–6 are highlighted in green.

Table S2. Matrix of integrals obtained by batch integration of the 135 ROIs defined with rNMR on the ^{1}H-^{13}C HSQC spectra.

Table S3. Matrix of integrals obtained after binning the ^{1}H spectra.
